# Zinc Oxide Nanoparticles Promote Apoptosis of Granulosa Cell Through Autophagy-Associated Suppression of Notch3 Signaling

**DOI:** 10.3390/antiox15101269

**Published:** 2026-10-02

**Authors:** Ziqi Wang, Yifan Wang, Guangyu Liu, Jing Lv, Hong Tang, Zhongliang Jiang

**Affiliations:** 1 Key Laboratory of Animal Genetic, Breeding and Reproduction in Shaanxi Province, College of Animal Science and Technology, Northwest Agriculture and Forestry University, Yangling 712100, China; qzw1121@nwafu.edu.cn (Z.W.); yifanwang.924@nwafu.edu.cn (Y.W.); guangyuliu@nwafu.edu.cn (G.L.); lvjing96@nwafu.edu.cn (J.L.); 2 Xinjiang Academy of Agricultural and Reclamation Sciences, Shihezi 832000, China; urblockhead@163.com

**Keywords:** zinc oxide nanoparticles, granulosa cells, autophagy, apoptosis, Notch3 signaling, ovarian toxicity

## Abstract

Zinc oxide nanoparticles (ZnO NPs) are increasingly used as feed additives and antimicrobial alternatives in livestock production, but their potential reproductive toxicity remains insufficiently understood. Autophagy and apoptosis are central regulators of follicular atresia and granulosa cell fate, but whether ZnO NPs disrupt these processes in granulosa cells and how they interact mechanistically remain unclear. In this study, we tested whether ZnO NPs couple autophagy to apoptosis in mouse granulosa cells (GCs) through Notch3 signaling. Primary GCs were exposed to ZnO NPs and analyzed via viability assays, RT-qPCR, immunoblotting, and RNA-seq, with pharmacological perturbation of autophagy, apoptosis, and Notch signaling. ZnO NPs reduced viability and induced autophagic and apoptotic responses. The autophagy inhibitor 3-methyladenine (3-MA) partially attenuated ZnO NPs-induced autophagy-associated alterations, improved viability, and attenuated apoptosis. Conversely, benzyloxycarbonyl-Val-Ala-Asp-fluoromethyl ketone (Z-VAD-FMK) reduced apoptosis and partially modulated autophagy-associated markers but did not fully reverse the ZnO NPs-induced autophagic response. Transcriptomics identified altered Notch signaling, and ZnO NPs reduced NOTCH3, the NOTCH3 intracellular domain, and HES1. 3-MA partially restored this axis, linking autophagy-associated alterations to Notch3 signaling suppression. Consistent with a protective role for Notch signaling, the γ-secretase inhibitor DAPT aggravated ZnO NPs-induced viability loss and apoptosis. Together, these pharmacological interventions support a model in which ZnO NPs-induced autophagic alterations contribute to apoptosis through suppression of Notch3 signaling, while apoptosis inhibition only partially modulates the autophagic response. This autophagy–Notch3–apoptosis regulatory network provides a framework for evaluating the ovarian toxicity of ZnO NPs.

## 1. Introduction

Zinc oxide nanoparticles (ZnO NPs) are widely used in biomedical products, food packaging, cosmetics, and livestock production because of their distinctive physicochemical and antimicrobial properties [1,2]. In animal husbandry, they are being developed as alternatives to conventional antibiotics, with potential benefits for animal health and production [3,4]. For example, conventional ZnO has been incorporated into basal broiler diets at 60 mg/kg feed [5]. Additionally, dietary supplementation with porous and nanosized ZnO particles at 500 mg/kg has been shown to improve growth performance and intestinal barrier function in piglets. Their expanding use, however, has raised growing concerns regarding their biosafety [6]. ZnO NPs can enter the body through ingestion, inhalation, dermal exposure, or injection, and prolonged exposure has been associated with hepatic, renal, pulmonary, and reproductive injuries [7,8]. In female offspring, gestational exposure to ZnO NPs has been reported to increase oocyte DNA damage, diminish ovarian reserve, and reduce the number of primordial follicles [9]. Lactational exposure via tail vein injection of ZnO NPs at doses of 5–15 mg/kg may also lead to their accumulation in the mammary gland and subsequent transfer into milk, accompanied by disruption of the blood–milk barrier [10]. In vivo studies showed that tail vein administration of ZnO NPs (5–20 mg/kg) to pregnant mice significantly increased Zn^2+^ levels in the placenta and embryos, and another study showed that oral gavage of ZnO NPs at 34 mg/kg/day for 30 consecutive days significantly impaired prefrontal cortex function in mice [11,12]. ZnO NPs have been shown to impair testicular function by disturbing the blood–testis barrier and reducing sperm quality in males [13]. These findings indicate that the toxicological effects of ZnO NPs are complex and tissue-dependent, underscoring the need for systematic investigations into their effects on reproductive cells. However, the direct effects and underlying mechanisms of ZnO NPs in ovarian granulosa cells (GCs) remain incompletely defined.

Follicular atresia eliminates most mammalian ovarian follicles before maturation [14]. GC apoptosis has long been regarded as a principal driver of this process [15]. Apoptosis is a major mechanism underlying GC loss, whereas autophagy may exert either adaptive or injury-promoting effects depending on the cellular context and stress intensity. Importantly, autophagy has been shown to facilitate oxidative stress-induced apoptosis of GCs, indicating that crosstalk between these two processes may critically influence GC fate under stress conditions [16,17,18]. For example, autophagy regulates GC differentiation through WT1 degradation [19], whereas excessive autophagic activity has been associated with GC dysfunction in polycystic ovary syndrome [20]. Our previous work showed that ZnO NPs activate autophagy and disrupt hormone secretion in porcine GCs [21]. Other studies have indicated that autophagy can promote GC apoptosis [22,23]. Although these studies suggest that ZnO NPs can affect both autophagy and apoptosis, whether ZnO NPs-induced autophagy promotes or follows apoptosis of GCs, as well as how this relationship is regulated, remains unresolved.

Notch signaling is an evolutionarily conserved, contact-dependent pathway that mediates cell-to-cell communication through interactions between Notch receptors and membrane-bound ligands of the Jagged and Delta-like families [24]. Upon ligand binding, proteolytic cleavage releases the Notch intracellular domain, which translocates into the nucleus and regulates downstream target genes such as HES and HEY, thereby controlling cell fate determination, proliferation, differentiation, and survival and tissue homeostasis [25]. Increasing evidence indicates that Notch signaling is also closely connected to cellular stress responses, particularly apoptosis and autophagy [26,27,28]. In the ovary, Notch components are expressed in oocytes, GCs, theca cells, and the vasculature, and they participate in follicle assembly, follicular growth, GC proliferation, differentiation, steroidogenesis, and survival [29]. Disruption of Notch signaling has been reported to impair GC function and follicular development, suggesting that this pathway is essential for maintaining ovarian homeostasis [30]. However, the mechanisms by which Notch signaling contributes to ZnO NPs-induced granulosa cell injury remain unclear.

In the present study, we hypothesized that ZnO NPs-induced autophagic alterations contribute to apoptosis through suppression of Notch3 signaling and interact with apoptotic pathways during GC injury. To test this model, we combined dose and time response assessments of GC viability with molecular and imaging analyses of autophagy and apoptosis; reciprocal perturbation with 3-methyladenine (3-MA) and benzyloxycarbonyl-Val-Ala-Asp fluoromethyl ketone (Z-VAD-FMK); RNA sequencing (RNA-seq) pathway analysis; measurement of NOTCH3, the NOTCH3 intracellular domain (NICD3), and HES1; and pharmacological inhibition of Notch signaling with γ-secretase inhibitor IX (DAPT). These findings provide mechanistic insight into ZnO NPs mediated reproductive toxicity and suggest that the autophagy–Notch3 axis may represent a critical regulatory pathway in nanoparticle-induced GC injury.

## 2. Materials and Methods

### 2.1. Preparation and Characterization of ZnO NPs

ZnO NPs (#Z820772) were purchased from Macklin Biochemical Co., Ltd. (Shanghai, China). The characterization of ZnO NPs was conducted in our previous study [21]. Briefly, ZnO NPs were dispersed in double distilled water at 1 mg/mL, vortexed for 1 min and sonicated in an ice bath for 30 min before use. Particle characterization was reported previously [21]. Briefly, particle size was examined by transmission electron microscopy (HT7800; Hitachi, Tokyo, Japan), morphology by scanning electron microscopy (SEM, FEI, Nano SEM-450; Hillsboro, OR, USA), and hydrodynamic size and surface charge by dynamic light scattering (Malvern Panalytical, Malvern, UK).

### 2.2. Animals and Granulosa Cell Culture

Twenty 10-week-old female Kunming (KM) mice were purchased from Chengdu Dossy Experimental Animals Co., Ltd. (Chengdu, China). The mice were housed at the Laboratory Animal Center of Northwest A&F University under controlled conditions (26 ± 1 °C and 55 ± 10% relative humidity) with a 12 h light/dark cycle and ad libitum access to food and water and were acclimatized for one week before ovarian collection.

Ovaries were collected from female mice euthanized by cervical dislocation, and GCs were isolated by puncturing the ovarian follicles with a sterile needle. The isolated cells were washed three times with phosphate-buffered saline (PBS), centrifuged at 1000 rpm for 5 min, and cultured in DMEM/F12 medium supplemented with 10% fetal bovine serum (FBS) and 1% penicillin–streptomycin. After 48 h of culture, the adherent GCs were passaged once, and the resulting passage 1 cells were cultured for an additional 24 h before experimental treatment.

### 2.3. Experimental Design and Pharmacological Treatments

Dose- and time-response screening evaluated ZnO NPs at 0, 2, 4, 8 and 16 μg/mL for 6, 12, 18 and 24 h. On the basis of this screening, 8 μg/mL ZnO NPs for 24 h was selected for subsequent experiments. Mechanistic experiments compared untreated control cells with cells exposed to ZnO NPs, 3-MA (0.1 mM, GC10710, Glpbio, Montclair, CA, USA), Z-VAD-FMK (20 μM, GC12861, Glpbio, USA) or DAPT (20 μM, GC12942, Glpbio, USA) alone, or the corresponding inhibitor together with ZnO NPs, as appropriate to the assay.

### 2.4. Cell Viability Assays

Cell viability was measured with a Cell Counting Kit-8 (CCK-8; Beyotime, Shanghai, China). Briefly, 5 × 10^3^ cells/well were seeded into a 96-well plate, with outer wells filled with PBS to limit evaporation. After treatment, 10 μL CCK-8 reagent was added per well and cells were incubated at 37 °C for 2 h. Absorbance at 450 nm was measured with a microplate reader (Bio-Rad, Hercules, CA, USA). Live and dead cells were also visualized using a Calcein AM/propidium iodide (PI) kit (C2015L; Beyotime, China). After treatment, cells were incubated with staining solution for 30 min and imaged with an Olympus BX40 fluorescence microscope (Olympus Corporation, Tokyo, Japan).

### 2.5. Apoptosis Assays

Apoptosis was quantified with an Annexin V/PI apoptosis detection kit (Beyotime, China). After 24 h of treatment, cells were detached with 0.25% trypsin–EDTA, neutralized with culture medium and centrifuged at 1000 rpm for 6 min. Cells were resuspended in binding buffer and stained with Annexin V/PI according to the manufacturer’s instructions. Samples were acquired on a flow cytometer (BD Biosciences, Paramus, NJ, USA), and data were analyzed in FlowJo v10.9.0.

Apoptosis was also assessed with a one-step TUNEL apoptosis assay kit (C1086; Beyotime). Cells were washed twice with PBS, fixed in 4% paraformaldehyde for 20 min and permeabilized with 0.1% Triton X-100 for 5 min. Cells were incubated with TUNEL reaction mixture at 37 °C for 1 h in the dark, counterstained with DAPI (C1006; Beyotime) and imaged by fluorescence microscopy. TUNEL-positive cells were quantified in at least three randomly selected fields per group.

### 2.6. Determination of Autophagosomes

GCs were seeded in 24-well plates at a concentration of 5 × 10^5^ cells/well and treated for 24 h. Cells were fixed in 4% paraformaldehyde for 20 min and incubated with 0.05 mM monodansylcadaverine (MDC) and 1 μg/mL DAPI for 15 min at 37 °C in the dark. Images were acquired by fluorescence microscopy and analyzed in ImageJ v6.1. Five randomly selected fields were evaluated per sample, and the mean intensity of green MDC-positive puncta was used as a measure of MDC-labeled vacuolar structures.

### 2.7. Immunofluorescence

After 24 h of treatment, GCs were fixed in 4% paraformaldehyde for 10 min at room temperature, washed with PBS and incubated in immunostaining blocking solution (P0102, Beyotime) for 1 h. Cells were incubated overnight at 4 °C with antibodies against LC3A/B (AF5402, Affinity, Shanghai, China, 1:250) or NOTCH3 (AF7548, Affinity, China, 1:300). After three washes with PBST, cells were incubated with CoraLite594-conjugated Goat Anti-Rabbit IgG(H + L) (SA00013-4, Proteintech, Wuhan, China, 1:500) or CoraLite488-conjugated Goat Anti-Mouse IgG(H + L) (SA00013-1, Proteintech, 1:500) secondary antibody for 1 h at room temperature in the dark. Nuclei were counterstained with DAPI where indicated, and fluorescence images were acquired using identical settings across groups.

### 2.8. Immunoblotting

GCs were seeded in six-well plates and treated for 24 h. Total protein was extracted in ice-cold RIPA buffer containing PMSF (ST505; Beyotime) and a protease inhibitor mixture (78430; Thermo Fisher Scientific, Waltham, MA, USA). Protein concentration was measured with a BCA assay kit (P0010S, Beyotime). Equal amounts of protein (20 μg) were resolved by 12% SDS–PAGE and transferred to PVDF membranes. Membranes were blocked with QuickBlock Western Blocking Buffer for 1 h at room temperature and incubated overnight at 4 °C with primary antibodies. Primary antibodies were directed against GAPDH (600041-Ig, Proteintech, 1:50,000), ATG7 (PA5-85388, Invitrogen, Carlsbad, CA, USA, 1:1000), ATG5 (DF6010, Affinity, 1:1000), ATG3 (AF7587, Affinity, 1:1000), BECN1 (AF5128, Affinity, 1:1000), SQSTM1 (PA5-20839, Invitrogen, 1:1000), LC3A/B (AF5402, Affinity, 1:1000), CYCS (AF1046, Affinity, 1:1000), BCL2 (MA5-11757, Invitrogen, 1:1000), BAX (MA5-14003, Invitrogen, 1:1000), CASP9 (MA5-53738, Invitrogen, 1:1000), CASP3 (AF6311, Affinity, 1:1000), cleaved CASP3 (AF7022, Affinity, 1:1000), NOTCH3 (AF7548, Affinity, 1:1000), NICD3 (YC0202, Immunoway, San Jose, CA, USA, 1:1000) and HES1 (YM8626, Immunoway, 1:2000). Membranes were washed three times with TBST and incubated for 1 h at room temperature with HRP-conjugated anti-mouse (SA00001-2, Proteintech, 1:5000) or anti-rabbit (SA00001-1, Proteintech, 1:5000) secondary antibody diluted in QuickBlock buffer. Bands were detected using enhanced chemiluminescence reagent (WBKLS0100, Millipore, Burlington, MA, USA), visualized with a ChemiDoc XRS+ system (Bio-Rad) and quantified in ImageJ. Representative blots were selected from at least three independent experiments, and target protein abundance was normalized to GAPDH.

### 2.9. Reverse Transcription Quantitative Polymerase Chain Reaction (RT-qPCR)

GCs were seeded in six-well plates and treated for 24 h. Total RNA was extracted with TRIzol reagent (Tiangen, Beijing, China) according to the manufacturer’s instructions, and cDNA was synthesized with HiScript II Q RT SuperMix for qPCR with gDNA wiper (Vazyme, Nanjing, China). The concentration and purity of the RNA and cDNA were assessed with a NanoDrop 2000 spectrophotometer (Thermo Fisher Scientific). RT-qPCR was performed with ChamQ SYBR qPCR Master Mix (Vazyme, China) in 20 μL reactions. Cycling comprised 95 °C for 30 s, followed by 40 cycles of 95 °C for 10 s and 60 °C for 20 s. Melting curve analysis was used to assess amplification specificity. *Gapdh* served as the reference gene, and relative expression was calculated using the 2^−ΔΔCt^ method. The primer sequences used for RT-qPCR are listed in Table 1.

### 2.10. RNA-Seq and Bioinformatic Analysis

Total RNA was extracted from control and ZnO NPs-treated GCs with TRIzol reagent, and RNA concentration and purity were measured with a NanoDrop 2000 spectrophotometer. Library preparation and transcriptome sequencing were performed by LC-Bio Technology (Hangzhou, China). Polyadenylated RNA was enriched with oligo(dT) magnetic beads and fragmented, followed by first- and second-strand cDNA synthesis, end repair, A tailing, adaptor ligation and PCR amplification. Libraries were sequenced in paired-end mode on an Illumina NovaSeq 6000 platform (Illumina, San Diego, CA, USA). Raw reads were processed with fastp to remove adaptor sequences, duplicate reads and low-quality reads. Clean reads were aligned to the *Mus musculus* reference genome with HISAT2, and transcripts were assembled and quantified with StringTie v2.1.4. Gene abundance was expressed as fragments per kilobase of transcript per million mapped reads (FPKM). Differential expression was analyzed with edgeR in R. Differentially expressed genes were defined as fold change > 1.5 and *p* < 0.05. Gene Ontology (GO) and Kyoto Encyclopedia of Genes and Genomes (KEGG) pathway enrichment analyses were performed with DAVID. Three transcriptomic samples per group were analyzed.

The raw transcriptome sequencing data generated in this study have been deposited in the China National Center for Bioinformation (CNCB), under BioProject accession number CRA048072.

### 2.11. Statistical Analysis

Experiments included at least three technical and biological replicates, and data are presented as the mean ± s.e.m. Normality was assessed with the Shapiro–Wilk test, and non-normally distributed data were logarithmically transformed before analysis. Statistical analyses were performed in GraphPad Prism 9.5, with *p* < 0.05 and *p* < 0.01 used as significance thresholds.

## 3. Results

### 3.1. ZnO NPs Induce Autophagy and Apoptosis in GCs

**Figure 1:**
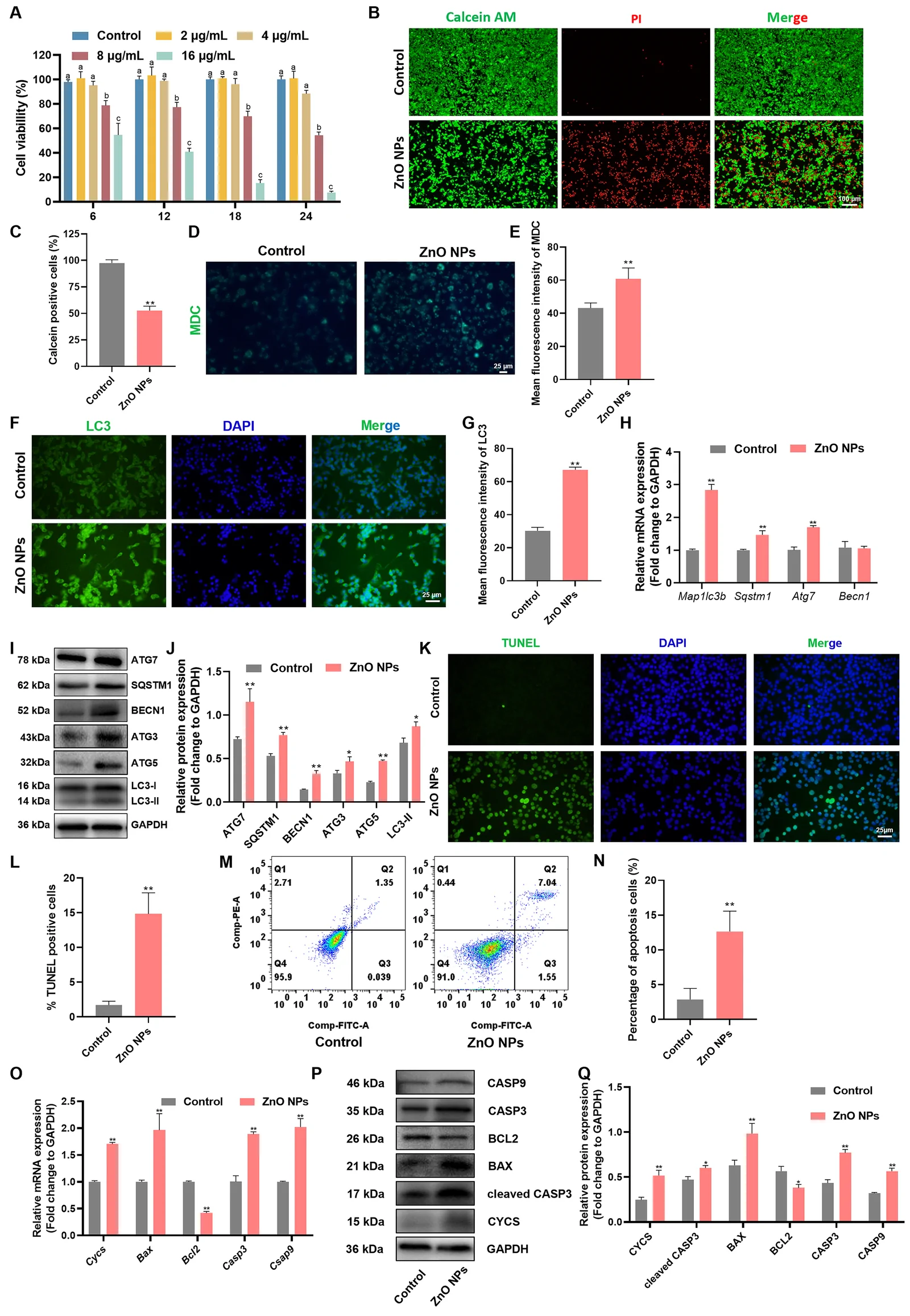
ZnO NPs induce autophagy and apoptosis of GCs. (**A**) CCK-8 analysis of GCs viability after exposure to ZnO NPs. (**B**,**C**) Representative Calcein AM/PI live/dead staining and quantification of the ratio of Calcein AM-positive to PI-positive cells. (**D**,**E**) MDC staining and quantification of mean MDC fluorescence intensity. (**F**,**G**) Representative LC3 immunofluorescence and quantification of LC3 fluorescence intensity. (**H**) RT-qPCR analysis of autophagy-related genes. (**I**,**J**) Immunoblotting of autophagy-related proteins with densitometric analysis. Densitometric quantification of LC3-II was normalized to GAPDH. (**K**,**L**) Representative TUNEL staining and percentage of TUNEL-positive cells. (**M**,**N**) Annexin V/PI flow cytometry and quantification of total apoptotic cells. In panel M, the black lines indicate the quadrant boundaries, and the dot colors represent event density. (**O**) RT-qPCR analysis of apoptosis-related genes. (**P**,**Q**) Immunoblotting and densitometric analysis of apoptosis-related proteins. Scale bars, 100 μm (**B**) and 25 μm (**D**,**F**,**K**). Data are mean ± s.e.m. from at least three independent experiments. Values marked by different lowercase letters differ at *p* < 0.05; * *p* < 0.05 and ** *p* < 0.01, as indicated.

Details of the characterization of ZnO nanoparticles have been reported in our previous study [21]. We first defined the ZnO NPs exposure condition for the mechanistic experiments. CCK-8 screening across 0, 2, 4, 8, and 16 μg/mL and 6, 12, 18, and 24 h showed a concentration- and time-dependent loss of GC viability (Figure 1A). Calcein AM/PI imaging likewise showed fewer viable cells, more PI-positive cells, and a lower ratio of Calcein AM-positive to PI-positive cells after ZnO NPs exposure (Figure 1B,C). On the basis of the screen, 8 μg/mL for 24 h was used in subsequent ZnO NPs experiments, providing a condition that produced a clear cellular response without the near-complete viability loss observed at the highest concentration. ZnO NPs increased monodansylcadaverine (MDC) fluorescence, as observed via fluorescence microscopy (Figure 1D,E). LC3 immunofluorescence was also altered after exposure (Figure 1F,G). At the transcript level, *Map1lc3b*, *Sqstm1*, and *Atg7* increased, whereas *Becn1* showed no clear change (Figure 1H). Immunoblotting showed increased ATG7, SQSTM1, BECN1, ATG3, ATG5, and LC3-II abundance (Figure 1I,J). Together, the increased MDC signal and coordinated changes in LC3-II and autophagy-related proteins, including ATG3, ATG5, ATG7, BECN1, and SQSTM1, indicate a ZnO NPs-induced autophagic response involving the autophagy-associated machinery in GCs. ZnO NPs concurrently increased TUNEL-positive cells and the Annexin V/PI-defined apoptotic population (Figure 1K–N). The *Cycs*, *Bax*, *Casp3*, and *Casp9* transcripts increased, whereas Bcl2 decreased (Figure 1O). Consistent with these changes, immunoblotting showed higher CYCS, BAX, CASP3, cleaved CASP3, and CASP9 abundance, together with lower BCL2 abundance (Figure 1P,Q). These findings indicate a ZnO NPs-induced autophagy-related response; however, they do not establish increased autophagic flux or the involvement of a specific upstream initiation pathway.

### 3.2. 3-MA Attenuates Autophagy-Associated Alterations and Partially Restores Viability

**Figure 2:**
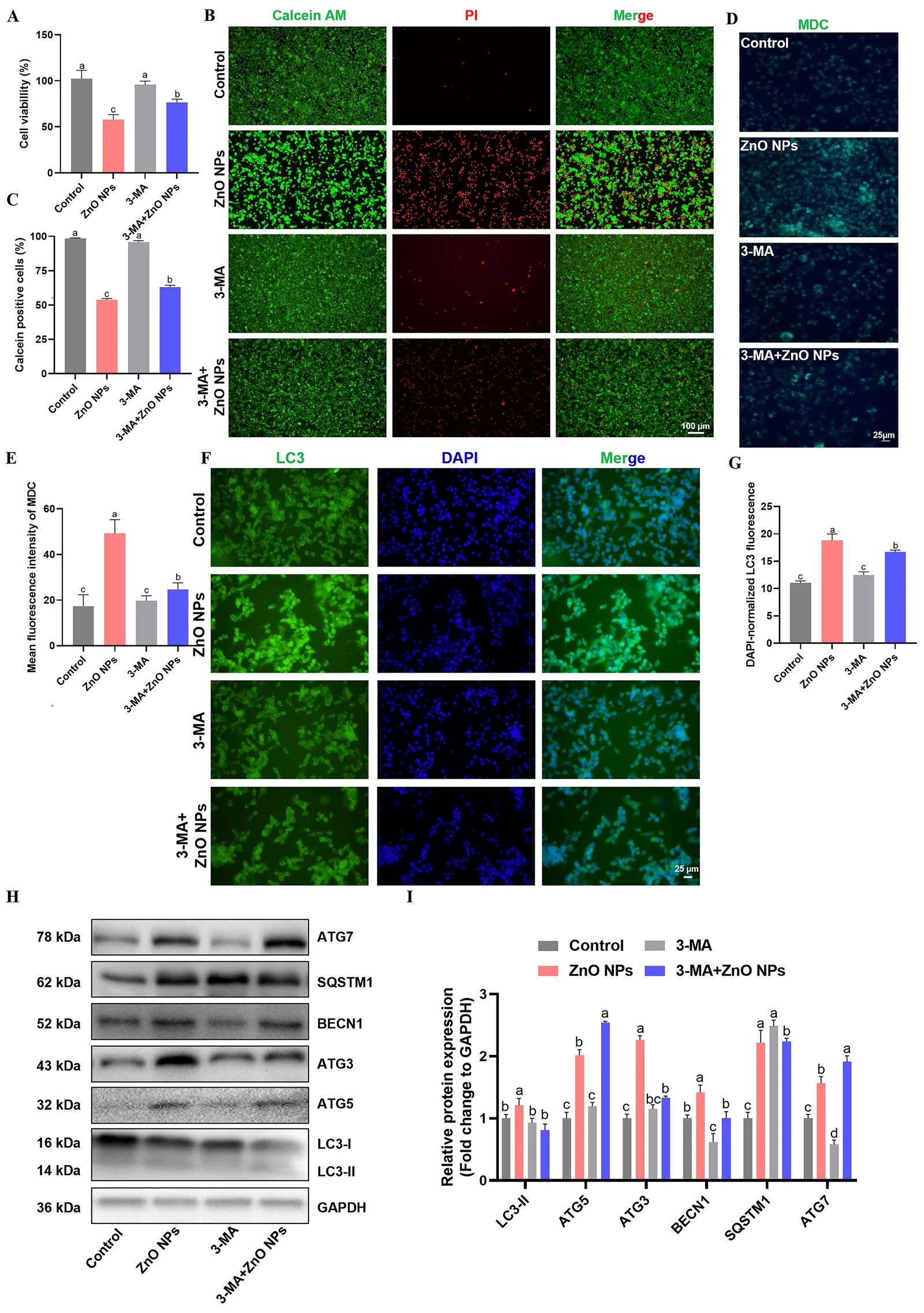
3-MA attenuates autophagic alterations and improves GC viability. GCs were treated with ZnO NPs (8 μg/mL), 3-MA (0.1 mM), or both for 24 h. (**A**) Cell viability measured by CCK-8 assay. (**B**,**C**) Representative Calcein AM/PI live/dead staining and quantification of the ratio of Calcein AM-positive to PI-positive cells. (**D**,**E**) Representative MDC staining and quantification of mean fluorescence intensity. (**F**,**G**) Representative LC3 immunofluorescence and quantification of LC3 fluorescence intensity. (**H**,**I**) Immunoblotting of autophagy-related proteins with densitometric analysis. Densitometric quantification of LC3-II was normalized to GAPDH. Scale bars, 100 μm (**B**) and 25 μm (**D**,**F**). Data are mean ± s.e.m. from at least three independent experiments. Values marked by different lowercase letters differ at *p* < 0.05.

To determine whether the autophagic response contributed to ZnO NPs-induced injury, GCs were co-treated with ZnO NPs and 0.1 mM 3-MA for 24 h. Relative to ZnO NP treatment alone, 3-MA co-treatment increased CCK-8 viability and improved the calcein AM/PI live/dead profile, although the combined treatment group did not fully return to the control state (Figure 2A–C). This recovery indicates that autophagy contributed to the loss of viability. 3-MA also reduced the ZnO NPs-induced increase in MDC fluorescence (Figure 2D,E) and attenuated the increase in LC3 immunofluorescence (Figure 2F,G). Immunoblotting further showed that, in the combined treatment group, following co-treatment with 3-MA and ZnO NPs, LC3-II, ATG3, and BECN1 expression showed an alleviating trend, whereas ATG5, ATG7, and SQSTM1 did not display obvious recovery and showed a partial increasing trend (Figure 2H,I). Together, these results indicate that 3-MA partially attenuated ZnO NPs-induced autophagy-associated alterations and partially restored GC viability.

### 3.3. Attenuation of Autophagy-Associated Alterations Reduces Apoptosis

**Figure 3:**
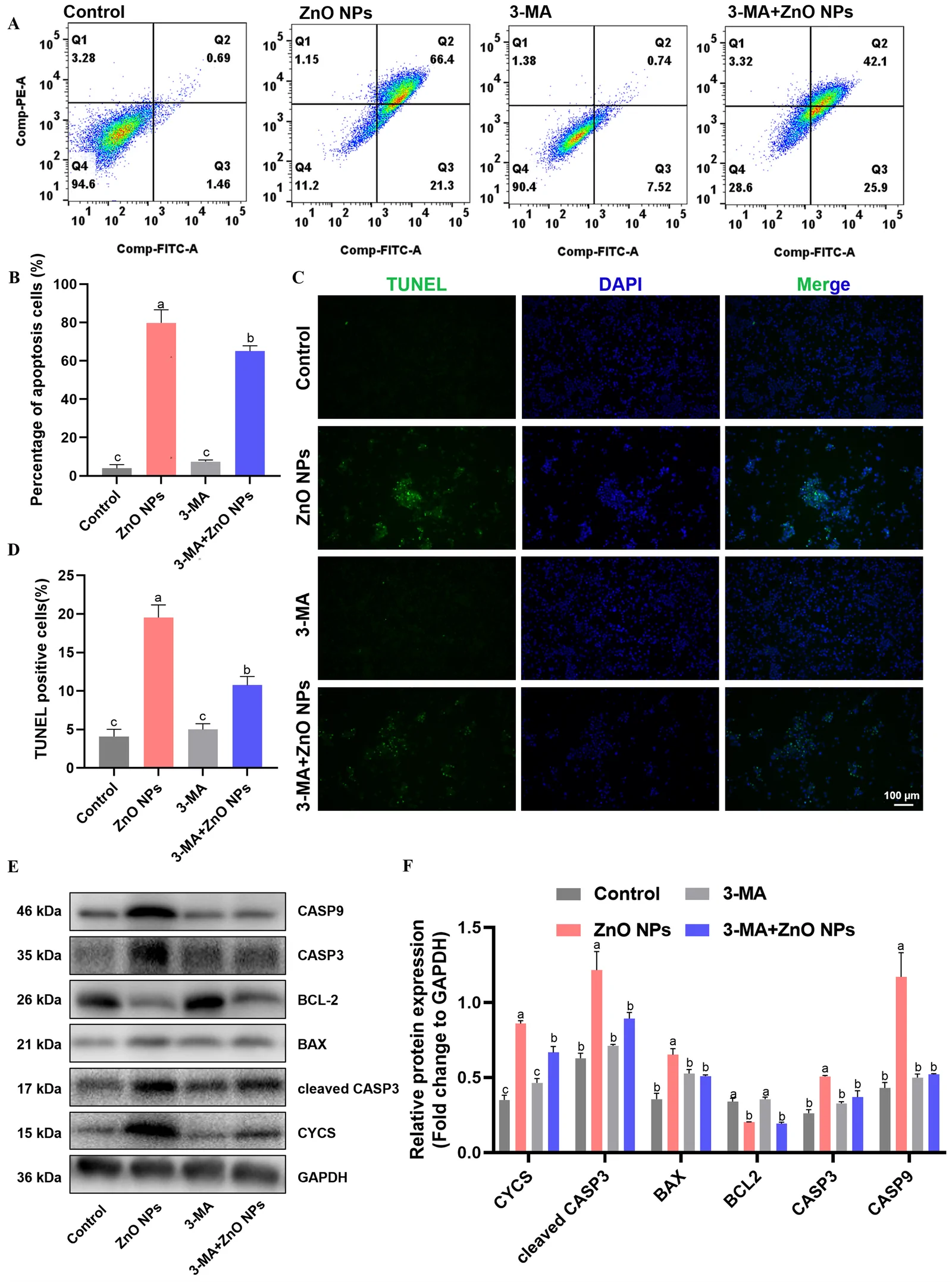
Attenuation of autophagy-associated alterations reduces apoptosis in GCs. GCs were treated with ZnO NPs (8 μg/mL), 3-MA (0.1 mM), or both for 24 h. (**A**,**B**) Annexin V/PI flow cytometry and quantification of total apoptotic cells. In panel A, the black lines indicate the quadrant boundaries, and the dot colors represent event density. (**C**,**D**) Representative TUNEL staining and percentage of TUNEL-positive cells. (**E**,**F**) Immunoblotting and densitometric analysis of apoptosis-related proteins. Scale bar, 100 μm (**C**). Data are mean ± s.e.m. from at least three independent experiments. Values marked by different lowercase letters differ at *p* < 0.05.

We next tested whether inhibiting autophagy altered the apoptotic response to ZnO NPs. Co-treatment with 0.1 mM 3-MA and ZnO NPs for 24 h reduced the Annexin V/PI-defined apoptotic population relative to treatment with ZnO NPs alone (Figure 3A,B). It also reduced TUNEL staining and the proportion of TUNEL-positive cells (Figure 3C,D), showing that attenuation of autophagy-associated alterations was accompanied by reduced apoptosis, as indicated by two independent cellular readouts. At the protein level, compared with the ZnO NPs group, 3-MA co-treatment attenuated the increases in CYCS, BAX, CASP3, cleaved-CASP3, and CASP9 expression, whereas BCL2 expression remained unchanged (Figure 3E,F). The molecular response was consistent with the flow cytometric and TUNEL endpoints. Together, these data support a model in which the ZnO NPs-induced autophagic response contributes to apoptotic injury in GCs.

### 3.4. Z-VAD-FMK Inhibits Apoptosis and Restores Viability

**Figure 4:**
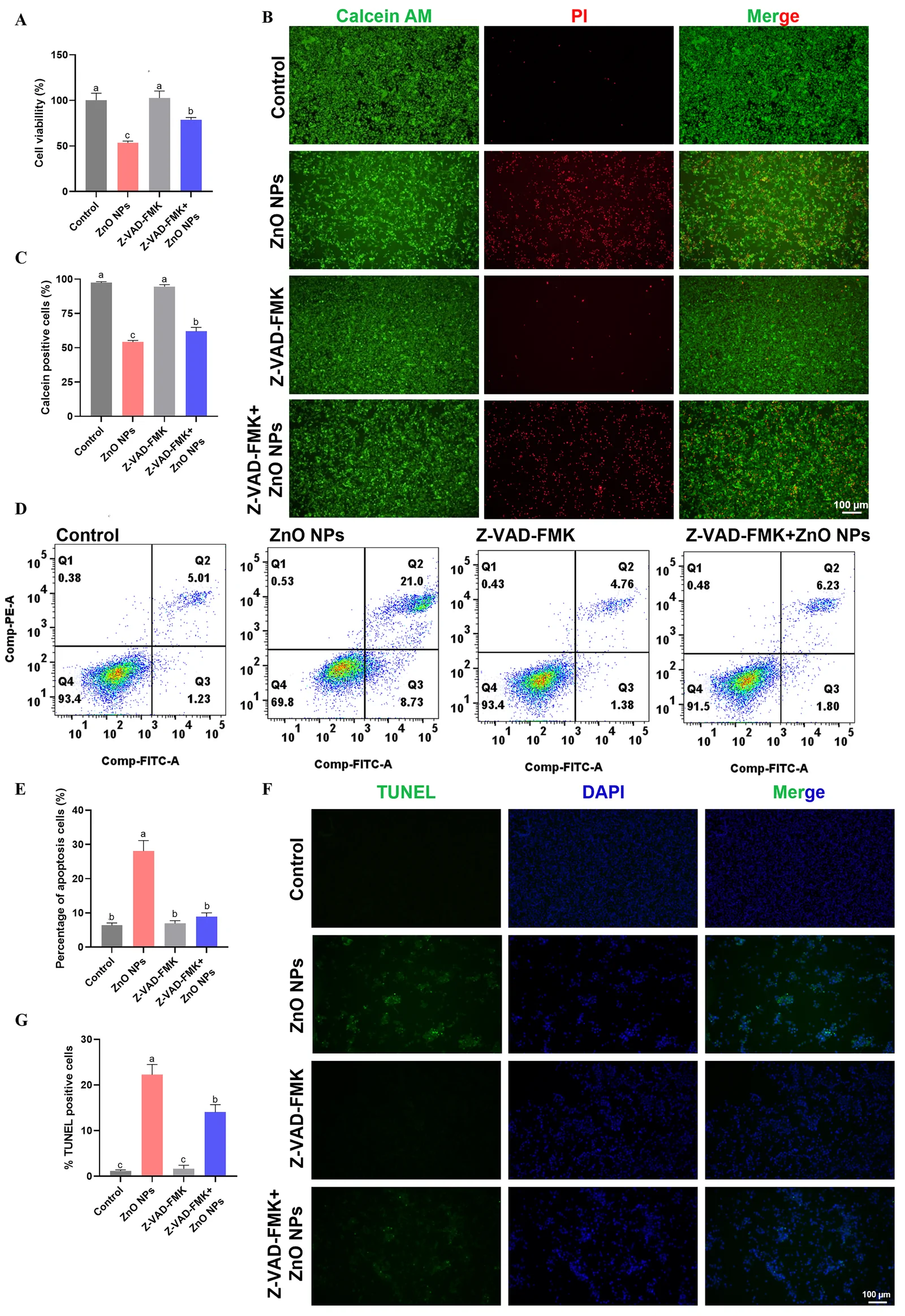
Z-VAD-FMK-mediated apoptosis inhibition restores viability of GCs. GCs were treated with ZnO NPs (8 μg/mL), Z-VAD-FMK (20 μM), or both for 24 h. (**A**) Cell viability measured by CCK-8 assay. (**B**,**C**) Representative Calcein AM/PI live/dead staining and quantification of the ratio of Calcein AM-positive to PI-positive cells. (**D**,**E**) Annexin V/PI flow cytometry and quantification of total apoptotic cells. In panel D, the black lines indicate the quadrant boundaries, and the dot colors represent event density. (**F**,**G**) Representative TUNEL staining and percentage of TUNEL-positive cells. Scale bars, 100 μm (**B**,**F**). Data are mean ± s.e.m. from at least three independent experiments. Values marked by different lowercase letters differ at *p* < 0.05.

To test the contribution of apoptosis to the loss of cell viability, GCs were co-treated with ZnO NPs and 20 μM Z-VAD-FMK for 24 h. Z-VAD-FMK co-treatment increased CCK-8 viability relative to ZnO NPs treatment alone (Figure 4A). Calcein AM/PI imaging likewise showed an improved live/dead profile (Figure 4B,C), indicating that suppression of apoptosis preserved cell viability. Z-VAD-FMK reduced the ZnO NPs-induced Annexin V/PI-positive apoptotic population (Figure 4D,E) and suppressed TUNEL staining and TUNEL positivity (Figure 4F,G). Together, these endpoints show that apoptosis makes a substantial contribution to ZnO NPs-induced GC injury and that Z-VAD-FMK preserves viability by inhibiting apoptosis.

### 3.5. Apoptosis Inhibition Partially Attenuates ZnO NPs-Induced Autophagic Alterations in GCs

**Figure 5:**
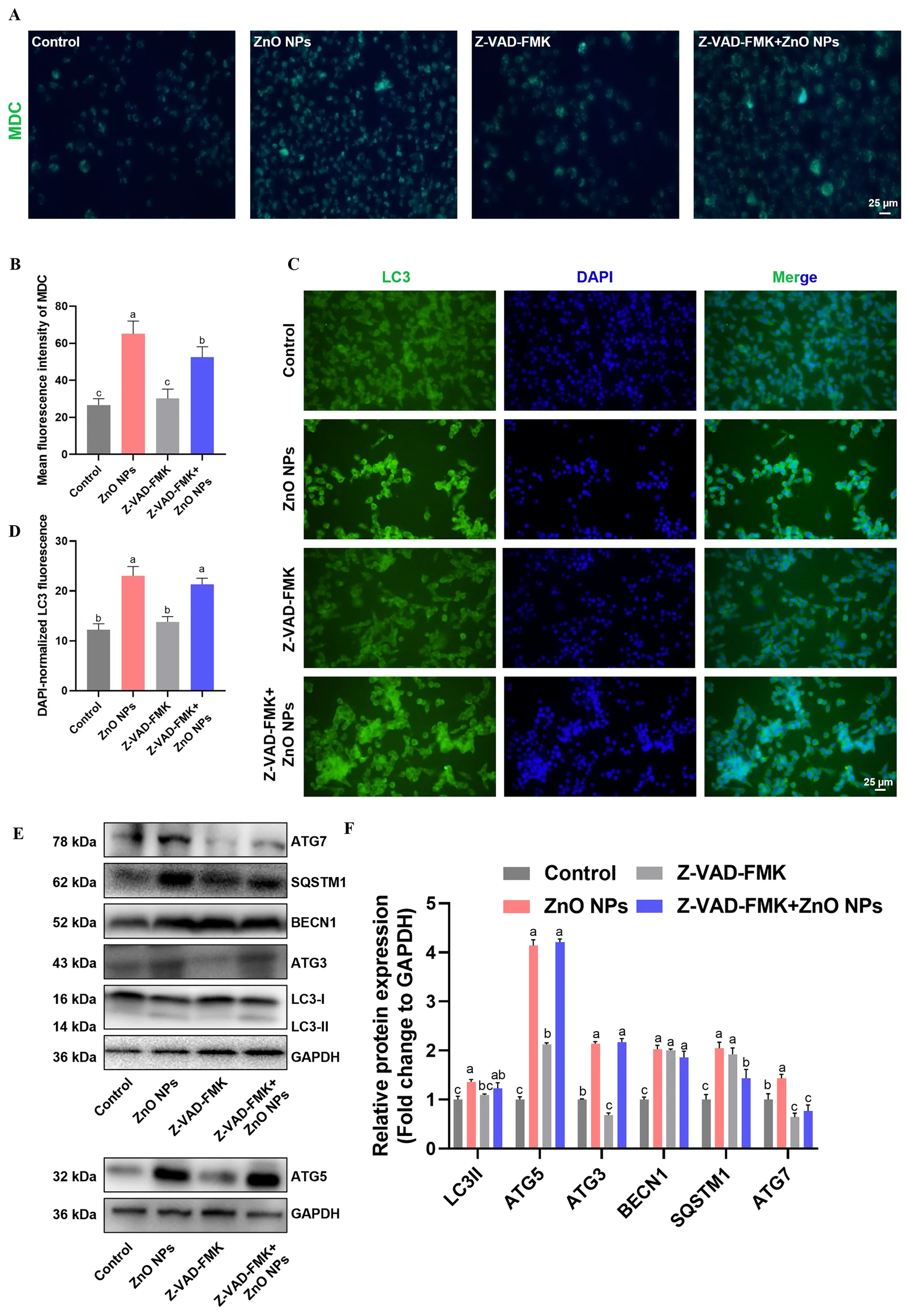
Z-VAD-FMK partially attenuates ZnO NPs-induced autophagic alterations in GCs. GCs were treated with ZnO NPs (8 μg/mL), Z-VAD-FMK (20 μM), or both for 24 h. (**A**,**B**) Representative MDC staining and quantification of mean fluorescence intensity. (**C**,**D**) Representative LC3 immunofluorescence and quantification of LC3 fluorescence intensity. (**E**,**F**) Immunoblotting of autophagy-related proteins with densitometric analysis. Densitometric quantification of LC3-II was normalized to GAPDH. Scale bars, 25 μm (**A**,**C**). Data are mean ± s.e.m. from at least three independent experiments. Values marked by different lowercase letters differ at *p* < 0.05.

We then examined whether inhibition of apoptosis influenced the ZnO NPs-induced autophagic response. GCs were co-treated with ZnO NPs and 20 μM Z-VAD-FMK for 24 h. Z-VAD-FMK partially attenuated the ZnO NPs-induced increase in MDC staining and mean MDC fluorescence intensity (Figure 5A,B). However, LC3 immunofluorescence also remained elevated in the Z-VAD-FMK plus ZnO NPs group (Figure 5C,D). Immunoblotting showed that co-treatment with Z-VAD-FMK and ZnO NPs slightly reduced ZnO NPs-induced LC3-II elevation; however, this change did not reach statistical significance. Z-VAD-FMK treatment significantly reduced the expression of SQSTM1 and ATG7, while BECN1, ATG5 and ATG3 levels were not significantly altered compared with the ZnO NPs group (Figure 5E,F). Notably, Z-VAD-FMK treatment alone also altered the expression of SQSTM1 and BECN1, suggesting that Z-VAD-FMK may itself influence selected components of the autophagic machinery. However, these changes were not accompanied by consistent alterations in other autophagy-related markers, suggesting that Z-VAD-FMK selectively affects specific components of the autophagic machinery rather than inducing a broad autophagic program under the present experimental conditions. Together with the protective effects of 3-MA, these findings indicate that ZnO NPs-induced autophagic alterations contribute to apoptotic injury, while the selective effects of Z-VAD-FMK on SQSTM1, ATG7, and BECN1 suggest a complex reciprocal interaction between autophagy and apoptosis.

### 3.6. Transcriptomics Identifies Notch Signaling

**Figure 6:**
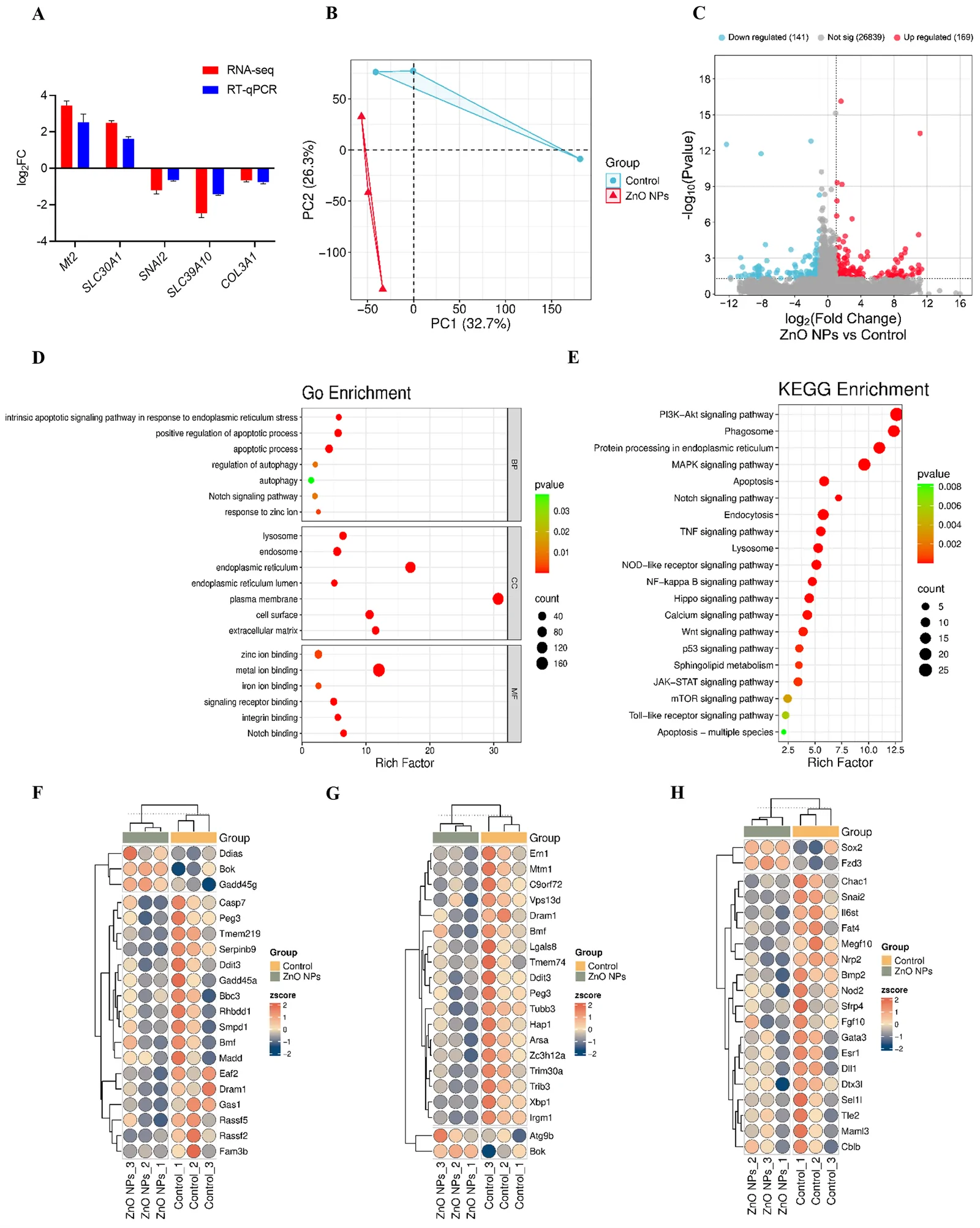
Transcriptomic profiling identifies Notch signaling as a potential regulator of ZnO NPs-induced autophagy alterations and apoptosis in GCs. GCs were treated with ZnO NPs (8 μg/mL) for 24 h. (**A**) Comparison of RNA-seq and RT-qPCR log_2_ fold changes for genes in ZnO NPs-treated GCs relative to controls. (**B**) Principal component analysis (PCA) of control and ZnO NPs-treated transcriptomes. (**C**) Volcano plot of differentially expressed genes in ZnO NPs-treated versus control GCs. (**D**) Gene Ontology (GO) enrichment analysis of biological process (BP), cellular component (CC) and molecular function (MF) categories. (**E**) Kyoto Encyclopedia of Genes and Genomes (KEGG) pathway enrichment analysis. (**F**–**H**) Heatmaps of genes related to apoptosis (**F**), autophagy (**G**) and Notch signaling (**H**). Three samples per group are shown, labeled Control-1−3 and ZnO NPs-1−3. Differentially expressed genes were defined by fold change > 1.5 and *p* < 0.05. Values are shown as mean ± s.e.m.

GCs were treated with ZnO NPs for 24 h, and RNA-seq was used to identify pathways linking ZnO NPs-induced autophagy to apoptosis. RNA-seq and RT-qPCR showed concordant directions of change for *Mt2*, *Slc30a1*, *Snal2*, *Slc39a10*, and *Col3a1*, supporting the expression trends detected via transcriptomic profiling (Figure 6A). Principal component analysis separated the control and ZnO NPs-treated samples (Figure 6B). At the reported threshold of fold change > 1.5 and *p* < 0.05, 718 genes were downregulated, 331 were upregulated, and 24,513 were classified as nonsignificant (Figure 6C). These data establish a broad transcriptional response to ZnO NPs exposure. GO enrichment included terms related to Notch signaling, regulation of apoptosis, cellular responses, and zinc ion biology (Figure 6D). KEGG analysis likewise identified Notch signaling, together with apoptosis, lysosome, and other enriched pathways (Figure 6E). Heatmaps showed coordinated expression changes in apoptosis- and autophagy-related gene sets and in the gene set annotated as Notch-related in the transcriptomic analysis (Figure 6F–H). Collectively, the enrichment analyses identified Notch signaling as a candidate molecular link between ZnO NPs-induced autophagy and apoptosis.

### 3.7. Autophagy-Associated Notch3 Suppression Contributes to Apoptosis in GCs

**Figure 7:**
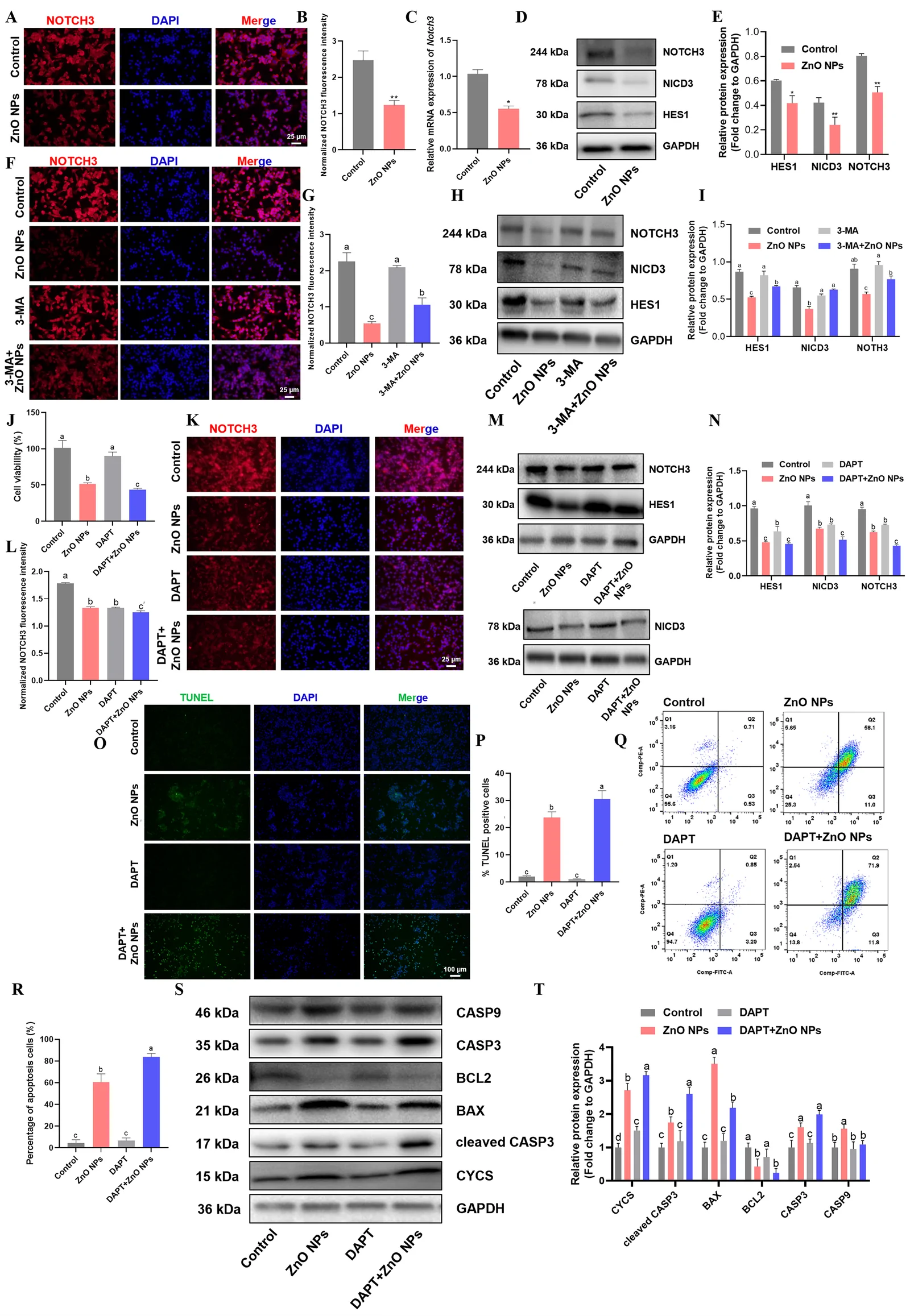
Autophagy-associated Notch3 suppression contributes to apoptosis in GCs. GCs were treated with ZnO NPs (8 μg/mL), DAPT (20 μM), or both for 24 h. (**A**,**B**) Representative NOTCH3 immunofluorescence in control and ZnO NPs-treated GCs, and quantification of mean NOTCH3 fluorescence intensity. (**C**) RT-qPCR analysis of *Notch3* mRNA. (**D**,**E**) Immunoblotting and densitometric analysis of NOTCH3, NICD3 and HES1. (**F**,**G**) Representative NOTCH3 immunofluorescence and quantification after treatment with ZnO NPs, 3-MA, or both. (**H**,**I**) Immunoblotting and densitometric analysis of NOTCH3, NICD3 and HES1 in the same groups. (**J**) CCK-8 analysis of cell viability after treatment with ZnO NPs, DAPT, or both. (**K**,**L**) Representative NOTCH3 immunofluorescence and quantification after DAPT treatment. (**M**,**N**) Immunoblotting and densitometric analysis of NOTCH3, NICD3 and HES1 after DAPT treatment. (**O**,**P**) Representative TUNEL staining and percentage of TUNEL-positive cells in the same four DAPT groups. (**Q**,**R**) Annexin V/PI flow cytometry and quantification of total apoptotic cells. In panel Q, the black lines indicate the quadrant boundaries, and the dot colors represent event density. (**S**,**T**) Immunoblotting and densitometric analysis of apoptosis-related proteins in the same four groups. Scale bars, 25 μm (**A**,**F**,**K**) and 100 μm (**O**). Data are mean ± s.e.m. from at least three independent experiments. Values marked by different lowercase letters differ at *p* < 0.05; * *p* < 0.05 and ** *p* < 0.01, as indicated.

**Figure 8:**
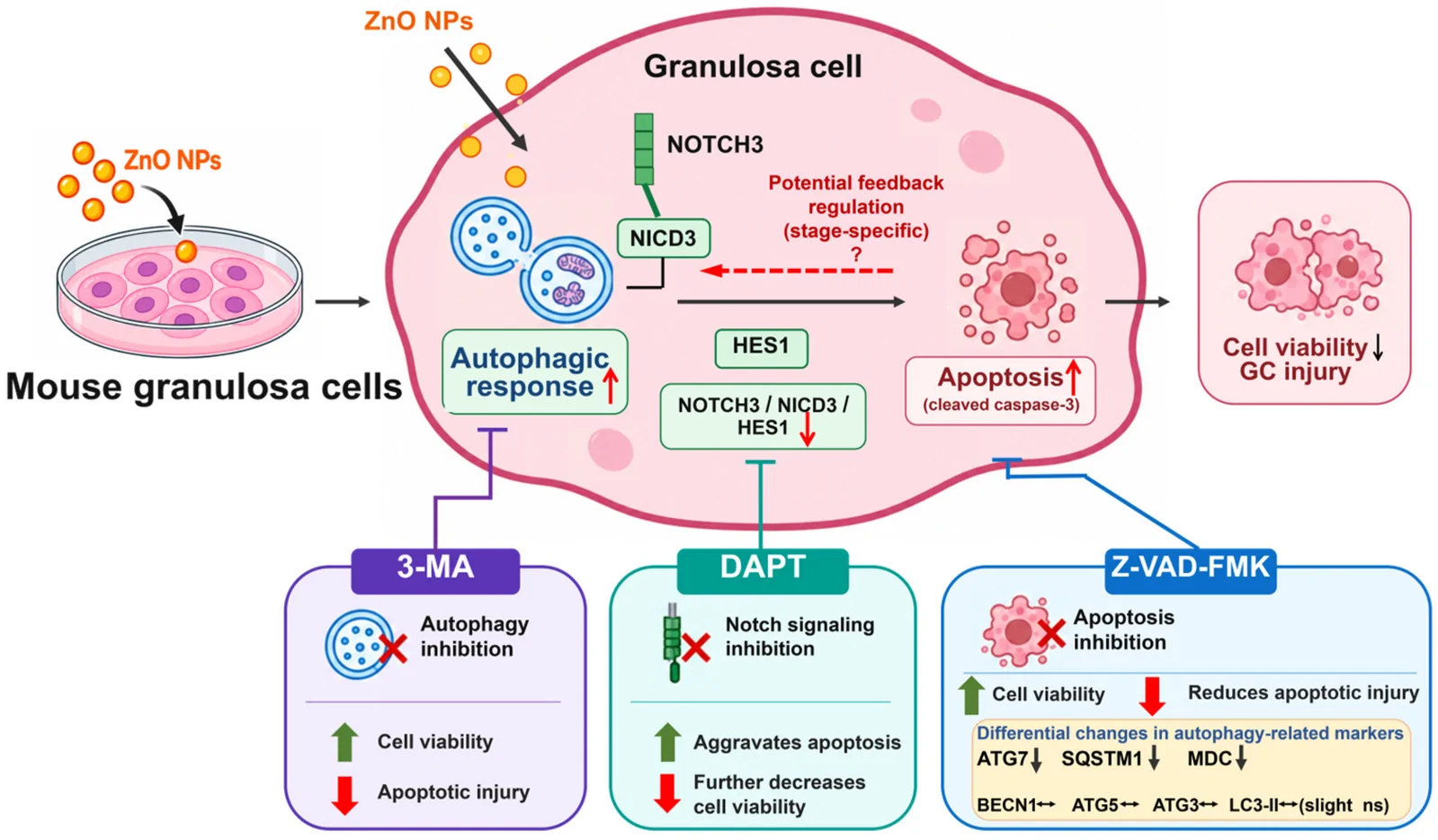
Schematic model of the proposed mechanism by which ZnO NPs promote apoptosis of granulosa cells through autophagy-associated suppression of Notch3 signaling. Solid arrows indicate the proposed regulatory relationships supported by the present findings, whereas the red dashed arrow indicates a potential feedback relationship suggested by the observed changes in this study, for which the underlying mechanism remains to be determined. Different colored lines are used to distinguish the three pharmacological interventions (3-MA, DAPT, and Z-VAD-FMK). Upward and downward arrows indicate increases and decreases, respectively.

We next tested the Notch3 axis identified via transcriptomic profiling. ZnO NPs reduced NOTCH3 immunofluorescence intensity and *Notch3* mRNA abundance (Figure 7A–C). Immunoblotting further showed lower NOTCH3, NICD3, and HES1 abundance after ZnO NPs exposure (Figure 7D,E). Thus, ZnO NPs reduced *Notch3* at the mRNA and protein levels and lowered the protein abundance of NICD3 and HES1. Autophagy-associated partially reversed this suppression. Compared with ZnO NP treatment alone, 3-MA co-treatment partially restored NOTCH3 immunofluorescence (Figure 7F,G) and partially recovered the protein levels of NOTCH3, NICD3, and HES1 (Figure 7H,I). These results link ZnO NPs-induced autophagy-associated alterations to the suppression of Notch3 signaling. To test whether the pharmacological inhibition of γ-secretase-dependent Notch signaling alters cell injury, GCs were co-treated with ZnO NPs and 20 μM DAPT for 24 h. DAPT co-treatment further reduced the CCK-8 viability readout relative to ZnO NPs treatment alone (Figure 7J). It also further reduced NOTCH3 immunofluorescence and produced a lower combined treatment profile for NOTCH3, NICD3, and HES1 than ZnO NPs treatment alone (Figure 7K–N). The combined perturbation therefore reinforced suppression of the Notch3 axis and loss of viability. DAPT co-treatment increased TUNEL positivity and the Annexin V/PI-defined apoptotic population compared to ZnO NPs treatment alone (Figure 7O–R). At the protein level, compared with the ZnO NPs group, DAPT co-treatment further increased the expression of CYCS, cleaved-CASP3, and CASP3, while decreasing the expression of BAX and CASP9. BCL2 expression was also increased following DAPT co-treatment. Notably, BAX expression showed an opposite trend, decreasing in the DAPT + ZnO NPs group compared with the ZnO NPs group. DAPT treatment alone also reduced BAX expression, suggesting a potential sensitivity of BAX expression to DAPT treatment. Nevertheless, the overall results indicate that DAPT co-treatment enhanced ZnO NPs-induced apoptosis in GCs (Figure 7S,T). Importantly, the enhanced apoptotic response following pharmacological inhibition of NOTCH signaling is consistent with our hypothesis that suppression of the NOTCH3/NICD3/HES1 axis contributes to ZnO NPs-induced apoptosis. Although BAX, CASP9, and BCL2 showed divergent protein-expression patterns, the increases in TUNEL positivity, Annexin V/PI-defined apoptosis, CYCS, cleaved CASP3, and CASP3 collectively indicate an enhanced apoptotic phenotype following DAPT co-treatment. Taken together, these findings support a model in which the ZnO NPs-induced autophagic response contributes to the suppression of the NOTCH3/NICD3/HES1 axis, thereby facilitating apoptotic signaling and loss of GC viability. A schematic summary of the proposed autophagy–Notch3–apoptosis axis is presented in Figure 8.

## 4. Discussion

ZnO NPs are used in food, biomedical, and livestock applications, raising concern about potential reproductive toxicity [31]. Reproductive effects have been reported in both sexes, but the mechanism of GC injury remains incompletely resolved [32,33,34,35].

Previous studies have linked ZnO NPs exposure to ovarian oxidative stress, follicular injury, impaired oocyte development, and apoptosis [36,37]. Our previous work further showed that ZnO NPs activate autophagy and disrupt cellular function in porcine GCs through ROS-dependent mechanisms [21]. The present study investigated the functional relationship between autophagy and apoptosis, identifying Notch3 signaling as a potential molecular link between these processes. The principal finding is that ZnO NPs-induced autophagic alterations contribute to apoptotic progression in GCs and are associated with suppression of the NOTCH3/NICD3/HES1 signaling axis, while pharmacological perturbation suggests functional crosstalk between autophagy and apoptosis. This provides a mechanistic framework connecting nanoparticle-induced cellular stress to a signaling pathway that is directly relevant to GC survival and follicular homeostasis.

The biological effects of ZnO NPs vary with particle properties, exposure conditions, and cellular context. Reported outcomes include disrupted embryonic development, reproductive endocrine changes, inflammation, and altered redox homeostasis and gene expression [33,38]. ZnO NPs exposure has been found to impair mouse preimplantation embryonic development by disrupting zygotic genome activation and transcriptional reprogramming [39]. These context-dependent toxic responses contrast with the beneficial use of ZnO nanomaterials in imaging and drug delivery [40]. In female reproductive models, ZnO NPs exposure has been associated with ovarian cell apoptosis in mice [41] and induces oxidative stress, while mitochondrial stress triggers autophagy and apoptosis, ultimately leading to oocyte death [42]. Our study reveals for the first time a potential link between ZnO NPs-induced autophagy and apoptosis in GCs involving suppression of Notch3 signaling.

Canonical autophagy initiation is regulated by upstream nutrient- and energy-sensing mechanisms, particularly the AMPK–mTORC1–ULK1 regulatory axis. Initiation and AMPK/ULK1-dependent autophagy has recently been demonstrated in stressed ovarian granulosa cells [43]. However, AMPK, mTOR, and ULK1 activation states were not examined in the present study. Therefore, our data define a ZnO NPs-induced autophagic response and its functional relationship with apoptosis and Notch3 signaling but do not identify the upstream kinase pathway responsible for autophagy initiation. Autophagy has complex functions in the ovary and can support cellular adaptation or contribute to cell injury depending on the magnitude and duration of the stress response [44,45]. In GCs, autophagy plays a dual role and may participate in oxidative stress-induced apoptosis [18], whereas ZnO NPs can also trigger autophagic cell death in other cellular systems [46]. These observations indicate that the biological consequence of autophagy cannot be inferred solely from increased autophagy-related markers. In the present study, 3-MA treatment partially attenuated ZnO NPs-induced autophagy-associated alterations, reduced apoptosis, and improved cell viability. Thus, under the present experimental conditions, the autophagic response was not merely a compensatory reaction to ZnO NP exposure but rather contributed to the progression of cellular injury. This interpretation is consistent with previous evidence that oxidative stress-associated autophagy can facilitate apoptosis of porcine GCs [18] and expands on our previous observation that ROS regulates ZnO NPs-induced autophagy in these cells [21].

BCL2 may represent one molecular interface between autophagy and apoptosis. ZnO NPs exposure reduced BCL2 while increasing BAX, CYCS, and caspase-associated apoptotic signaling. Although 3-MA attenuated several ZnO NPs-induced pro-apoptotic changes, BCL2 expression was not significantly restored. Therefore, the protective effect of 3-MA appears to involve modulation of multiple apoptotic components rather than restoration of BCL2 alone. These findings support an association between the autophagic response and mitochondrial apoptotic signaling, but do not establish BCL2 as the direct molecular mediator linking these processes [47,48]. A reduction in BCL2 may therefore favor mitochondrial apoptotic signaling by shifting the BCL2/BAX balance toward mitochondrial permeabilization while simultaneously altering the regulation of autophagy. Nevertheless, the present findings do not demonstrate that BCL2 itself directly mediates the transition from autophagy to apoptosis, and this relationship requires targeted mechanistic validation. As a broad-spectrum apoptosis inhibitor, Z-VAD-FMK was further applied in the present study to clarify the relationship between apoptosis and autophagy. Inhibition of apoptosis reduced ZnO NPs-induced apoptotic injury and improved cell viability but produced differential changes in autophagy-related markers. Compared with the ZnO NPs group, BECN1, ATG5, and ATG3 levels remained largely unchanged, indicating that Z-VAD-FMK did not uniformly alter the abundance of proteins associated with autophagy initiation, nucleation, and autophagosome formation. In contrast, ATG7 protein expression decreased, whereas LC3-II showed only a slight, non-significant reduction, with no appreciable change in LC3 fluorescence. These findings suggest that apoptosis inhibition may not substantially alter the steady-state abundance of LC3-positive autophagic structures but may influence selected components associated with ATG7-dependent autophagosome formation [49]. In addition, MDC staining was markedly reduced. Given that MDC is an acidotropic dye that preferentially labels acidic autophagy-related vesicular compartments rather than serving as a specific marker of early autophagosomes, the reduced MDC signal may reflect alterations in late-stage autophagic compartments, potentially involving autophagosome maturation, autophagosome-lysosome fusion, and/or subsequent acidification [50]. Meanwhile, SQSTM1/p62 protein levels were decreased. As SQSTM1/p62 is an autophagic substrate that is incorporated into autophagosomes and subsequently degraded within autolysosomes, its reduction is not consistent with a simple model of complete late-stage autophagic blockade, which is generally associated with SQSTM1/p62 accumulation [50,51]. Nevertheless, decreased SQSTM1/p62 levels alone cannot be interpreted as evidence of enhanced autophagic flux, because its steady-state abundance is also influenced by transcriptional regulation, protein synthesis, and alternative degradation pathways [50,52]. Thus, the concomitant reductions in MDC staining and SQSTM1/p62 may reflect complex alterations in late-stage autophagic processing rather than a uniform inhibition of autophagy. Collectively, these differential changes suggest non-uniform modulation of autophagy-associated components following apoptosis inhibition rather than uniform activation or suppression of the autophagic program. The observation that Z-VAD-FMK alone altered BECN1 and SQSTM1 expression further suggests that caspase inhibition itself may influence selected autophagy-associated pathways. However, because these changes were not accompanied by alterations in LC3-II, LC3 fluorescence, ATG5, or ATG3, they should not be interpreted as evidence of global autophagy activation. Nevertheless, these effects of Z-VAD-FMK alone should be considered when interpreting the autophagy-associated changes observed in the co-treatment group. The precise mechanisms underlying the selective regulation of BECN1 and SQSTM1 by Z-VAD-FMK require further investigation. Meanwhile, because LC3-II, SQSTM1/p62, and MDC represent primarily steady-state or indirect measures of autophagy, the present findings are insufficient to determine whether autophagic flux is actually altered. Combined with the protective effects of 3-MA against ZnO NPs-induced injury, these findings indicate that autophagic signaling contributes to apoptotic progression rather than being solely a consequence of apoptosis. Furthermore, under the present experimental conditions, both Z-VAD-FMK alone and Z-VAD-FMK co-treatment appeared to influence specific components of the autophagic machinery, further supporting a complex interplay between autophagy and apoptosis. Conversely, Z-VAD-FMK attenuated apoptosis but produced selective changes in several autophagy-related markers without uniformly reversing the ZnO NPs-induced autophagic response. Taken together, these observations support functional crosstalk between autophagy and apoptosis rather than two independent processes. However, the present data do not establish a strictly unidirectional or sequential relationship between these pathways. Instead, they suggest that autophagy contributes to the progression of ZnO NPs-induced apoptosis, while apoptosis may, in turn, modulate specific components of the autophagy-associated response. Further studies employing dynamic autophagic flux assays and stage-specific interventions are required to define the directionality and molecular mechanisms underlying this crosstalk.

The identification of Notch signaling by transcriptomic analysis provides a potential mechanism linking these two processes. Notch signaling is an important regulator of ovarian follicular development and GC proliferation, differentiation, and survival. Suppression of this pathway could therefore render GCs more susceptible to stress-induced injury. In the present study, ZnO NPs reduced NOTCH3, NICD3, and HES1, whereas 3-MA treatment partially restored this signaling axis. Studies have shown that increased autophagy can regulate Notch signaling by promoting turnover of the Notch intracellular domain [53]. Conversely, Notch3–mTOR signaling has also been implicated in the regulation of autophagy [54], indicating that communication between these pathways can be bidirectional and context-dependent. In our study, inhibition of autophagy partially restored NOTCH3, NICD3, and HES1 expression, suggesting that ZnO NP-induced autophagy contributes to the suppression of Notch3 signaling.

Further investigation using DAPT showed that Notch signaling may play a supportive role in GC survival. Co-treatment with DAPT and ZnO NPs inhibited γ-secretase-dependent Notch pathway activation, further suppressed the NOTCH3/NICD3/HES1 axis, and consequently reduced cell viability and increased apoptosis. This suggests a role for γ-secretase-dependent signaling in GC proliferation and apoptosis, consistent with previous reports [55,56], showing that Notch signaling supports ovarian GC development [57] and that reduced ovarian Notch activity is associated with altered apoptotic and steroidogenic regulation [56,58]. Thus, residual Notch signaling appears to serve a protective function during ZnO NPs-induced cellular stress. Importantly, the present study links this response to autophagy-associated alterations: 3-MA treatment partially restored Notch3 signaling, whereas further pharmacological suppression of Notch signaling aggravated cellular injury. This relationship provides the main mechanistic advance of the study and supports an autophagy–Notch3–apoptosis regulatory axis contributing to ZnO NPs-induced GC injury.

Several limitations should be considered. First, DAPT inhibits γ-secretase-dependent Notch activation but is not specific to Notch3; therefore, future studies using Notch3 knockdown or overexpression are needed to confirm its direct causal role. Second, ZnO NPs exposure was performed under nutrient-rich basal conditions without nutrient-starvation control. Nutrient deprivation is a well-established stimulus of canonical autophagy that activates the AMPK–ULK1 axis while relieving mTORC1-mediated inhibition of ULK1. The absence of such a control limits our ability to determine whether the autophagy-related changes induced by ZnO NPs involve canonical nutrient-sensing regulation of autophagy initiation. Moreover, the ULK1 initiation complex and its upstream AMPK/mTOR regulators were not directly examined, and dynamic autophagic flux assays were not performed. Therefore, the present data cannot distinguish whether the observed autophagy-related response primarily reflects enhanced autophagy initiation, altered autophagosome turnover, or impaired downstream autolysosomal processing, nor can they establish the involvement of the canonical AMPK/mTOR–ULK1 pathway. Future studies incorporating nutrient-starvation controls, direct assessment of AMPK/mTOR–ULK1 signaling, and dynamic autophagic flux assays using lysosomal inhibitors or tandem fluorescent LC3 reporters will be required to distinguish these possibilities. Third, transcriptomic data also revealed alterations in PI3K-Akt, Wnt, and related pathways. Whether these pathways interact with Notch3 to form a broader regulatory network during ZnO NPs-induced GC injury remains to be investigated.

Overall, our findings support a model in which ZnO NPs induce coordinated autophagic and apoptotic responses in GCs. Autophagic alterations contribute to apoptotic progression through suppression of the NOTCH3/NICD3/HES1 axis, while pharmacological inhibition of apoptosis partially modulates specific autophagy-associated markers, indicating crosstalk between these processes. This study also reveals a coordinated autophagy–Notch3–apoptosis signaling network that may help explain how ZnO NPs disrupt granulosa cell homeostasis and provides a mechanistic basis for further evaluation of their ovarian reproductive toxicity.

## 5. Conclusions

ZnO NPs induce coordinated autophagic and apoptotic responses in GCs, and autophagic alterations contribute to apoptosis through suppression of the NOTCH3/NICD3/HES1 signaling axis. Pharmacological perturbation with 3-MA and Z-VAD-FMK indicates that autophagy and apoptosis are interconnected rather than strictly unidirectional processes under the tested in vitro conditions. Restoration of the NOTCH3/NICD3/HES1 axis by 3-MA and aggravation of cellular injury by DAPT further support this mechanism.

## Figures and Tables

**Table 1:** Primers used for quantitative real-time PCR.

Gene	Primer Sequence (5′-3′)	RefSeq Accession No.
*Map1lc3b*	F: GAGGGGACCCTAACCCCATA	NM_026160.5
	R: TCGCTCTATAATCACCCGCC	
*S* *qstm1*	F: ACTGCTCAGGAGGAGACGAT	NM_011018.3
	R: CCGGGGATCAGCCTCTGTAG	
*A* *tg7*	F: CACCTGACAGTGAGGCTGGTA	NM_028835.5
	R: TTAAAGGGGGCGAACTGCAA	
*B* *ecn1*	F: AGGAACTCACAGGAGCCATT	NM_019584.4
	R: CTCCCCGATCAGAGTGAAGC	
*N* *otch3*	F: ACTCCTCCTCAGGGAGATGC	NM_008716.3
	R: GTGGGGTGAAGCCATCAGG	
*Mt2*	F: CGTGGGCTGTGCGAAGTGCTC	NM_008630.2
	R: AAAGGCTAGGCTTCTACATGGTC	
*Slc30a1*	F: CAACACCAGCAATTCCAACGGG	NM_009579.3
	R: CGCTTCCAGATTGTCAGACTCC	
*Snal2*	F: TCTGTGGCAAGGCTTTCTCCAG	NM_011415.3
	R: TGCAGATGTGCCCTCAGGTTTG	
*Slc39a10*	F: CTCACGTTCCTTGTTGCACTAGC	NM_172653.3
	R: TGTGAATGACCATGTCCGTGCG	
*Col3a1*	F: GACCAAAAGGTGATGCTGGACAG	NM_009930.2
	R: CAAGACCTCGTGCTCCAGTTAG	
*G* *apdh*	F: ACCCTTAAGAGGGATGCTGC	NM_008085.2
	R: CCCAATACGGCCAAATCCGT	

## Data Availability

The sequencing data generated in this study have been deposited in the Genome Sequence Archive (GSA) at the China National Center for Bioinformation (CNCB), https://www.cncb.ac.cn/ (accessed on 14 August 2026) under accession number CRA048072. The other original contributions presented in this study are included in the article.
